# The gp130/STAT3-endoplasmic reticulum stress axis regulates hepatocyte necroptosis in acute liver injury

**DOI:** 10.3325/cmj.2023.64.149

**Published:** 2023-06

**Authors:** Xia Li, Jie Wang, Ying Li, Wei He, Qi-Jiao Cheng, Xia Liu, De-Lin Xu, Zhi-Gang Jiang, Xue Xiao, Yi-Huai He

**Affiliations:** 1 Department of Infectious Diseases, Affiliated Hospital of Zunyi Medical University, Zunyi, Guizhou, China; 2 Department of General Medicine, Affiliated Hospital of Zunyi Medical University, Zunyi, Guizhou, China; 3 Department of Cell Biology, Zunyi Medical University, Zunyi, Guizhou, China; 4 School of Public Health, Zunyi Medical University, Zunyi, Guizhou, China; The first two authors contributed equally.

## Abstract

**Aim:**

To investigate the effect of the gp130/STAT3-endoplasmic reticulum (ER) stress axis on hepatocyte necroptosis during acute liver injury.

**Methods:**

ER stress and liver injury in LO2 cells were induced with thapsigargin, and in BALB/c mice with tunicamycin and carbon tetrachloride (CCl_4_). Glycoprotein 130 (gp130) expression, the degrees of ER stress, and hepatocyte necroptosis were assessed.

**Results:**

ER stress significantly upregulated gp130 expression in LO2 cells and mouse livers. The silencing of *activating transcription factor 6 (ATF6)*, but not of *ATF4*, increased hepatocyte necroptosis and mitigated gp130 expression in LO2 cells and mice. *Gp130* silencing reduced the phosphorylation of CCl_4_-induced signal transducer and activator of transcription 3 (STAT3), and aggravated ER stress, necroptosis, and liver injury in mice.

**Conclusion:**

ATF6/gp130/STAT3 signaling attenuates necroptosis in hepatocytes through the negative regulation of ER stress during liver injury. Hepatocyte ATF6/gp130/STAT3 signaling may be used as a therapeutic target in acute liver injury.

The liver is prone to injury by pathogens and toxins (1). Liver injury can induce compensative anti-injury responses, such as endoplasmic reticulum (ER) stress in hepatocytes, to reduce their degeneration and necrosis (2). These defense responses can mitigate the sensitivity of hepatocytes to injury stimuli (3). However, the mechanisms through which they modulate ER stress-related liver injury have not been clarified.

Necroptosis is a mode of programmed cell death, characterized by a rupture of cell membranes and release of cell contents (4). Although necroptosis shows morphological changes similar to necrosis, it is regulated by a specific molecular mechanism (5). Biochemical markers of necroptosis are the RIPK1-RIPK3 complex formation and mixed lineage kinase domain-like pseudokinase (MLKL) phosphorylation. Furthermore, the release of cell contents leads to pro-inflammatory cell death and hepatocyte injury (6). Therefore, effective control of necroptosis can reduce liver injury.

ER stress, activated by various physiological and pathological conditions (7), contributes to the pathogenesis of liver diseases (8,9). During ER stress, unfolded proteins accumulate in the ER lumen and bind to glucose-regulated protein 78 (GRP78), which promotes the activation of protein kinase R-like ER kinase (PERK) and inositol requiring enzyme 1 (IRE1), and the translocation of activating transcription factor 6 (ATF6) (10). ATF6 acts as a transcription factor and induces the expression of ER chaperone proteins (11). Overall, ER stress represses protein synthesis by activating PERK/eIF2α signaling (12), IRE1 endonuclease activity (13), and ER-associated degradation (ERAD) (14), as well as promotes cell survival. However, persistent or aberrant ER stress can induce cell injury, such as caspase-dependent cell apoptosis and caspase-independent necroptosis. ER stress mediates hepatocyte apoptosis by activating CHOP and caspase-12, so that the protein levels of CHOP and caspase 12 reflect the levels of ER stress.

Glycoprotein 130 (gp130) is a transmembrane protein crucial for acute-phase response and liver regeneration. Hepatocyte-specific *Gp130* knockout mice are prone to acute liver injury (15). Gp130 is a functional co-receptor used by several cytokines and mediators, including interleukin (IL)-6, IL-11 (16), IL-27 (17), ciliary neurotrophic factor (18), leukemia inhibitory factor, cardiotrophin-1, oncostatin-M (19), and cardiotrophin-like cytokine (20). Gp130 dimerization, promoted by IL-6 binding to its α-receptor subunit (glycoprotein 80, gp80) and the coupled gp130 (the β-receptor subunit), leads to the activation of the Janus kinases (JAK) and signal transducer and activator of transcription 3 (STAT3) signaling (21). The activated STAT3 enhances acute phase response, liver regeneration, hematopoietic cell proliferation and differentiation, as well as pathological processes such as inflammation and tumorigenesis (22). Furthermore, the dimerized gp130 can also activate mitogen-activated protein kinase (MAPK) and phosphatidylinositol 3-kinase (PI3K) (23). Inactivation of the IL-6/gp130 pathway in the liver can mitigate acute phase reaction (24). However, the role of the hepatocyte gp130-ER stress-necroptosis axis in liver injury is poorly understood.

In this study, we investigated the effect of ER stress on gp130 expression in mouse models of acute liver injury induced by tunicamycin (TM, an ER stress inducer) and carbon tetrachloride (CCl_4_), and in a cellular model of ER stress induced by thapsigargin (TG). In addition, we investigated the impact of *GP130* silencing on ER stress, necroptosis, and liver injury.

## Materials and methods

### Induction of ER stress *in vitro*

A human hepatocyte LO2 cell line was obtained from the Cell Bank of Type Culture Collection of the Chinese Academy of Sciences (Shanghai, China). The cells were cultured in RPMI-1640 supplemented with 10% fetal bovine serum, 100 units/mL of penicillin, and 100 μg/mL of streptomycin. To induce ER stress, LO2 cells were treated with TG (0.5 μmol/L, Sigma, St. Louis, MO, USA) for 24 or 48 h. TG is a ER stress inducer that disrupts intracellular calcium homeostasis in the ER membrane and inhibits its ability to fold and process proteins (25). One control group was untreated and the other was treated with vehicle dimethyl sulfoxide (DMSO).

### Transfection

LO2 cells (1.2 × 10^6^ cells/well) were cultured overnight in six-well plates. They were transfected with a control plasmid to induce the expression of scramble shRNA or the plasmid for the expression of *ATF4*- or *ATF6*-specific shRNA (Beijing Syngentech, Beijing, China) by using Lipofectamine 3000 (Thermofisher Scientific, Waltham, MA, USA) for 48 h. The shRNA sequences and their targets are shown in Table 1. Different cell groups were treated with vehicle control or TG for 24 h to induce ER stress (26). Accordingly, the cells were divided into the control (control shRNA + DMSO), *ATF4*-KD or *ATF6*-KD (*ATF4* or *ATF6* shRNA + DMSO; KD: knockdown), TG (control shRNA + TG), and *ATF4*-KD or *ATF6*-KD + TG group (*ATF4* or *ATF6* shRNA + TG).

**Table 1:** The shRNAs sequences and their targets

Insert content		shRNA sequence (5′ to 3′)
ATF4*	Target sequence	GGAGATCCAGTACCTGAAAGA
	*ATF4* shRNA	GGAGATCCAGTACCTGAAAGACGAA*TCTTTCAGGTACTGGATCTCC*
	Control shRNA	AAACGTGACACGTTCGGAGAACGAATTCTCCGAACGTGTCACGTTT
ATF6	Target sequence	GCAGGTCCTCCTGTTATTAGA
	*ATF6* shRNA	GCAGGTCCTCCTGTTATTAGACGAA*TCTAATAACAGGAGGACCTGC*
	Control shRNA	AAACGTGACACGTTCGGAGAACGAATTCTCCGAACGTGTCACGTTT

*ATF – activating transcription factor.

### Cell viability assay

The impact of ER stress and altered gene expression on LO2 cell viability was assessed with an MTS cell viability assay (Cell Titer 96^®^ AQueous One Solution Cell Proliferation assay, Promega, Madison, WI, USA) as per the manufacturer's protocol. In brief, different cell groups (1 × 10^5^ cells/well) were cultured in triplicate in 96-well plates at 37 °C for varying time periods. During the last three hours of culture, to each well we added 20 μL of MTS (3-(4, 5-dimethylthiazol-2-yl)-5-(3-carboxymethoxyphenyl)-2-(4-sulfophenyl)-2H-tetrazolium) solution. The absorbance of individual wells was recorded at 490 nm in a microplate reader (model 680; Bio-Rad, Hercules, CA, USA), and the viability of different cell groups at a specific time point was normalized to the untreated control group (27).

### Induction of liver injury in mice

Two hundred and sixteen male BALB/c mice (7-8 weeks old, 23 ± 3.0 g) were purchased from the Animal Center of Zunyi Medical University (Guizhou, China). They were randomized into 18 groups (12 mice per group) by using a simple random number table method. The mice were maintained in a specific pathogen-free facility with a constant temperature of 20-24 °C, 12-h light/dark cycle, and food and water *ad libitum*. The experimental protocol was approved by the Animal Care Use Committee of the Affiliated Hospital of Zunyi Medical University. All experimental procedures complied with the relevant guidelines and regulations.

To test ER stress and gp130 expression, the untreated control mice were injected intraperitoneally with phosphate buffer saline (PBS, 10 mL/kg, TM solvent) or olive oil (5 mL/kg, CCl_4_ solvent); the TM group with 2 mg/kg TM (Sigma, St. Louis, MO, USA) for 24 h or 48 h; and the CCl_4_ group with a 5 mL/kg-mixture containing 1 mL CCl_4_ (Sigma) and 4 mL olive oil, for 24 h or 48 h. TM is an ER stress inducer that hinders glycosylation of nascent proteins in the ER (28,29). CCl_4_ can induce liver injury through its direct effect and its metabolic products of free radicals. Particularly, CCl_3_, which is produced by the decomposition of CCl_4_, can cause lipid peroxidation, leading to cell membrane damage (30). Accordingly, the groups were as follows: untreated, 24 h TM and its solvent control, and 48 h TM and its solvent control (5 groups, total = 60 mice); untreated, 24 h CCl_4_ and its solvent control, and 48 h CCl_4_ and its solvent control (5 groups, total = 60 mice). The protocol was based on our preliminary study.

To determine the effect of *Atf6* or *Gp130* silencing on ER stress-induced hepatocyte injury, the BALB/c mice were divided into 8 groups (total 96 mice): control (control shRNA + olive oil), *Atf6*-KD or *Gp130*-KD (*Atf6* or *Gp130* shRNA + olive oil), CCl_4_ (control shRNA + CCl_4_), and *Atf6*-KD or *Gp130*-KD + CCl_4_ groups (*Atf6* or *Gp130* shRNA + CCl_4_) (Table 2). The mice were first injected intravenously with 1 × 10^10^ virions of a recombinant adeno-associated virus serotype 8 (rAAV8) that expressed control shRNA, *Atf6-specific* shRNA, or *Gp130-specific* shRNA (Beijing Syngentech, Table 3) in 100 μL of PBS. The same dose of virions was given two days later as a booster. Six weeks after boosting, the mice were injected intraperitoneally with vehicle or CCl_4_. Twenty-four and/or 36 hours later, the mice were anesthetized with carbon dioxide inhalation. Blood samples were collected, and the mice were euthanized. The liver tissues were dissected for subsequent experiments.

**Table 2:** Mice groups and treatment in the experiment conducted to assess the effect of *Atf6* or *Gp130* silencing on endoplasmic reticulum stress-induced hepatocyte injury *

Group		Treatment
ATF6	Control	Control shRNA + olive oil
	*Atf6*-KD	*Atf6* shRNA + olive oil
	*Atf6*-KD + CCl_4_	*Atf6* shRNA + CCl_4_
	CCl_4_	Control shRNA + CCl_4_
Gp130	Control	Control shRNA + olive oil
	*Gp130*-KD	*Gp130* shRNA + olive oil
	CCl_4_	Control shRNA + CCl_4_
	*Gp130*-KD + CCl_4_	*Gp130* shRNA + CCl_4_

*Abbreviations: ATF – activating transcription factor; CCl_4_ – carbon tetrachloride; Gp130 – glycoprotein 130; KD – knockdown.

**Table 3:** Recombinant AAV8 vectors for mice

Insert content		shRNA sequence (5′ to 3′)
ATF6	Target sequence	GCAGTCGATTATCAGCATACA
	*Atf6* shRNA	GCAGTCGATTATCAGCATACACGAA*TGTATGCTGATAATCGACTGC*
	Control shRNA	AAACGTGACACGTTCGGAGAACGAATTCTCCGAACGTGTCACGTTT
Gp130	Target sequence	GCTACATGCCCACCTATTATG
	*Gg130* shRNA	GCTACATGCCCACCTATTATG CGAACA*TAATAGGTGGGCATGTAGC*
	Control shRNA	CCTAAGGTTAAGTCGCCCTCGCCGAAGCGAGGGCGACTTAACCTTAGG

*ATF – activating transcription factor; Gp130 – glycoprotein 130.

### Western blotting

The liver tissues were homogenized in an immunoprecipitation assay lysis buffer (10 mg/mL, R0010, Solarbio, Beijing, China). After centrifugation, the relative levels of the studied proteins to the control β-actin in liver lysate samples were quantified by using Western blotting with 10% gels (31). The specific antibodies included mouse monoclonal antibodies (mAbs) against ATF6 (sc-166659, Santa Cruz Biotechnology, Dallas, TX, USA), actin (sc-376421, Santa Cruz Biotechnology), eIF2α (sc-133227, Santa Cruz Biotechnology), gp130 (sc-376280, Santa Cruz Biotechnology), C/EBP homologous protein (CHOP, ab11419, abcam, Cambridge, MA, USA), STAT3 (sc-8019, Santa Cruz Biotechnology), rabbit mAbs against ATF4 (11815, Cell Signaling Technology, Danvers, MA, USA), phosphorylated eIF2α (p-eIF2α, Ser51, 3398, Cell Signaling Technology), phosphorylated STAT3 (p-STAT3, 9145, Cell Signaling Technology), phosphorylated MLKL (p-MLKL, 37333S, Cell Signaling Technology), and MLKL (PA5-34733, ThermoFisher Scientific, Waltham, MA, USA), or rabbit polyclonal antibodies against caspase-12 (35965s, Cell Signaling Technology). The bound antibodies were detected with horseradish peroxidase-conjugated anti-mouse (sc-516102) or anti-rabbit IgG (sc-2357, both from Santa Cruz Biotechnology) and visualized with enhanced chemiluminescent reagents. Densitometric analysis was carried out with Quantity One software (Bio-Rad).

### Histochemical and immunohistochemical analysis

The paraffin-embedded liver tissue sections (5 μm) were routinely stained with hematoxylin and eosin (32) and scanned with a panoramic slice scanner (Pannoramic DESK/MIDI/250/1000, 3DHISTECH, Budapest, Hungary). The generated images were viewed with CaseViewer2.3 software (3DHISTECH). After background adjustment, the necrotic tissue areas were measured with Image-Pro Plus 6.0 software in three visual fields (33). The liver tissue sections were scored by two experienced pathologists using the Histology Activity Index-Knodell score in a blinded manner (34,35). Additionally, the liver tissue sections were stained with anti-gp130 antibody (sc-376280, 1:100; Santa Cruz Biotechnology) and visualized with the diaminobenzidine (DAB) reagent (PV-9002, ZSBIO, Beijing, China).

### Serum alanine aminotransferase and total bilirubin levels

The levels of serum alanine aminotransferase (ALT) and total bilirubin (TBil) were measured with a Beckman Coulter auto-analyzer (AU5800, Beckman Coulter, Brea, CA, USA) (36).

### Analysis of ATF6 binding to the GP130 promoter

The promoter sequence of human *GP130* gene at its transcription start site of 2000 bp was retrieved from the UCSC website (http://genome.ucsc.edu/index.html). Bioinformatic analysis (*https://jaspar.genereg.net/)* was used to identify the binding sites of ATF6 in the *GP130* promoter with a relative score of >0.75. The relative score of each binding site was based on the corresponding algorithm calculation. A higher score implied a greater likelihood of ATF6 binding (37).

### Statistical analysis

The normality of distribution was assessed with a one-sample Kolmogorov-Smirnov test. Data are presented as mean ± standard deviation (SD). The differences among the groups were assessed with a one-way analysis of variance (ANOVA) and a *post-hoc* least significant difference test. A *P*-value of <0.05 was considered statistically significant (38). The analysis was performed with SPSS 18.0 (SPSS, Chicago, IL, USA).

## Results

### ER stress upregulates gp130 expression in LO2 cells

**Figure 1:**
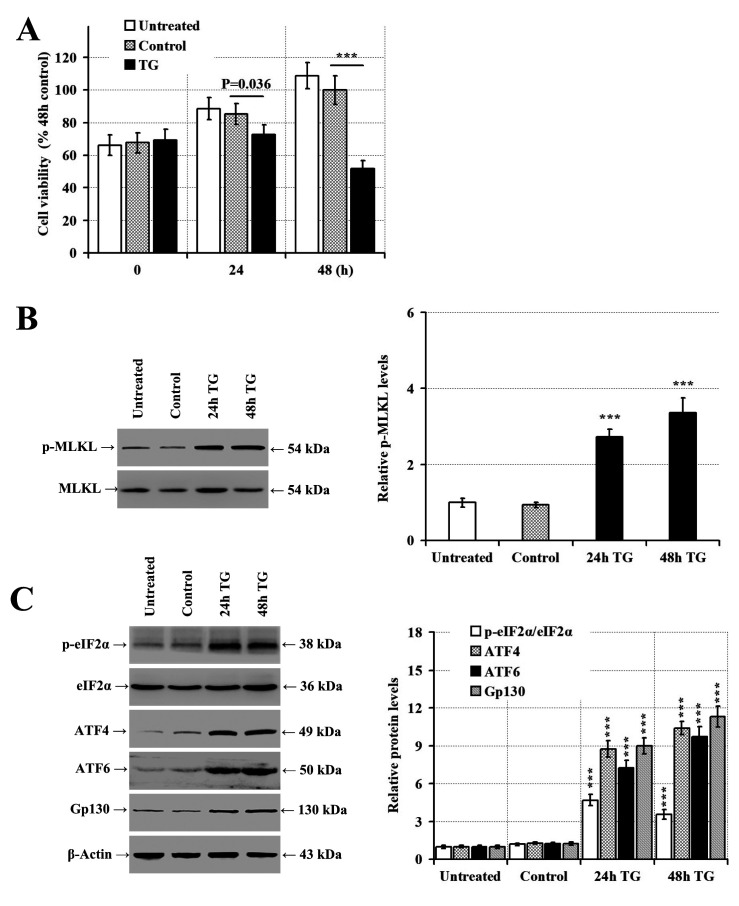
Endoplasmic reticulum (ER) stress upregulates glycoprotein 130 (gp130) expression in LO2 cells. (**A**) Analysis of LO2 cell viability by an MTS assay. LO2 cells were incubated with thapsigargin (TG) (0.5 μmol/L) or dimethyl sulfoxide (DMSO, control) for 24 or 48 h. (**B**) Western blot analyses of the relative levels of phosphorylated mixed lineage kinase domain-like pseudokinase (MLKL). (**C**) phosphorylated eukaryotic translation initiation factor 2α (eIF2α), activating transcription factor 4 (ATF4), ATF6, and gp130 protein at 24 h and 48 h post-TG incubation. Data are representative images or expressed as the mean ± standard deviation (SD) of each group from four independent experiments. ***P < 0.001 vs the control group or specified group.

Compared with vehicle treatment, TG treatment significantly reduced the viability of LO2 cells in a time-dependent manner (*P* = 0.036, and *P* < 0.001 at 24 and 48 h, respectively; Figure 1A). It also increased MLKL and eIF2α phosphorylation, as well as ATF4, ATF6, and gp130 protein expression (*P* < 0.001; Figure 1B and 1C, respectively). Hence, ER stress upregulated gp130 expression and necroptosis in hepatocytes.

### The silencing of ATF6, but not of ATF4, reduces gp130 expression in LO2 cells after inducing ER stress

**Figure 2:**
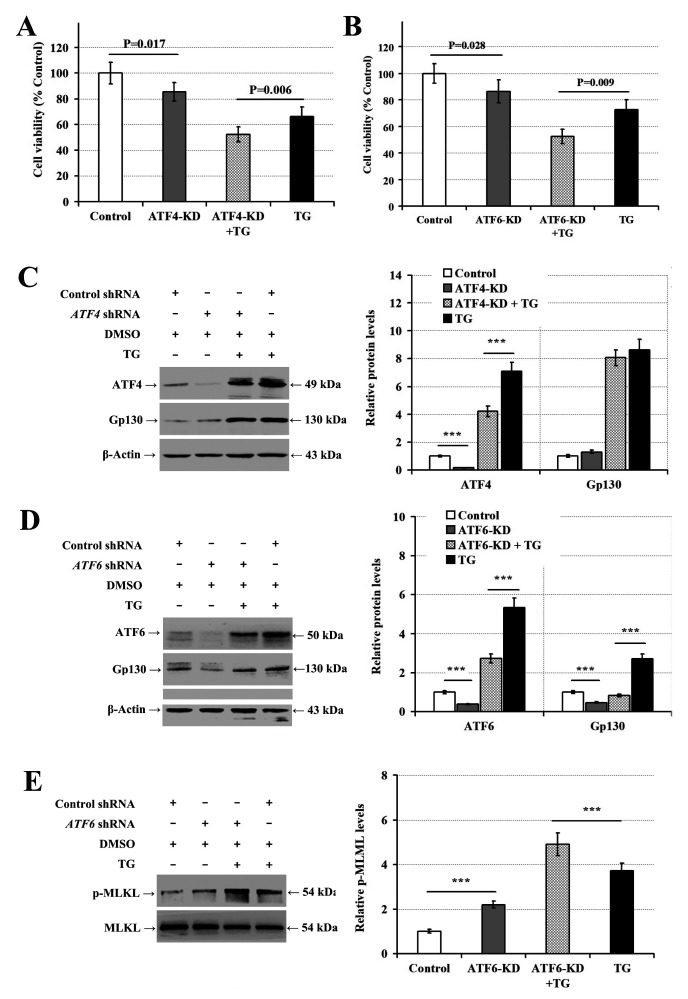
*Activating transcription factor 6* (*ATF6*) silencing mitigates thapsigargin (TG)-upregulated glycoprotein 130 (gp130) expression in LO2 cells. LO2 cells were pre-treated with *ATF4* shRNA, *ATF6* shRNA, or control shRNA for 48 h, and stimulated with TG (0.5 μmol/L) or dimethyl sulfoxide (DMSO, control) for 24 h. (**A**) The viability of *ATF4*- and (**B**) *ATF6*- shRNA-transfected LO2 cells as assessed with an MTS assay. (**C**) Western blot analyses of the relative levels of ATF4, ATF6, and gp130 protein expression in LO2 cells after transfection with *ATF4* shRNA or (**D**) *ATF6* shRNA. (**E**) Western blot analysis of the relative levels of mixed lineage kinase domain-like pseudokinase (MLKL) phosphorylation in different groups of LO2 cells. Data are representative images or expressed as mean ± standard deviation (SD) of each group from three separate experiments. ***P < 0.001 vs the specified group.

*ATF4* or *ATF6* silencing significantly decreased the viability of LO2 cells (*P* = 0.017 and *P* = 0.028, respectively) and further decreased the viability of TG-treated LO2 cells (*P* = 0.006 and *P* = 0.009, respectively; Figure 2A and B). Furthermore, it decreased ATF6 or ATF4 expression by 15%-60%. *ATF6*, but not *ATF4*, silencing significantly mitigated gp130 expression upregulated by TG (Figure 2C and D). This finding suggests that ER stress upregulates gp130 expression, partially depending on ATF6 expression. Moreover, *ATF6* silencing significantly increased the relative levels of MLKL phosphorylation in both control and TG-treated LO2 cells (*P* < 0.001; Figure 2E), a finding that suggests that ATF6 attenuates hepatocyte necroptosis. Bioinformatic analysis using JASPAR predicted two binding sites in the human *GP130* promoter for human ATF6, based on the relative profile score of ≥75% (Table 4). In conclusion, gp130 expression upregulated by ER stress may depend on ATF6 expression in LO2 cells.

**Table 4:** Prediction of human activating transcription factor (ATF) 6 binding to human glycoprotein 130 (*GP130*) promoter*

Score	Relative score	Start	End	Strand	Predicted sequence
5.173	0.775	48	61	+	ttgtgacatatcaa
3.198	0.750	491	504	-	cagccgggtcagca

*human-*GP130* promoter:>hg38_knownGene_ENST00000381298.7; range = chr5:55994964-55996963 5′pad = 0 3′pad = 0 strand = - repeatMasking = none.

### TM and CCl_4_ induce ER stress and upregulate gp130 expression in mouse hepatocytes

**Figure 3:**
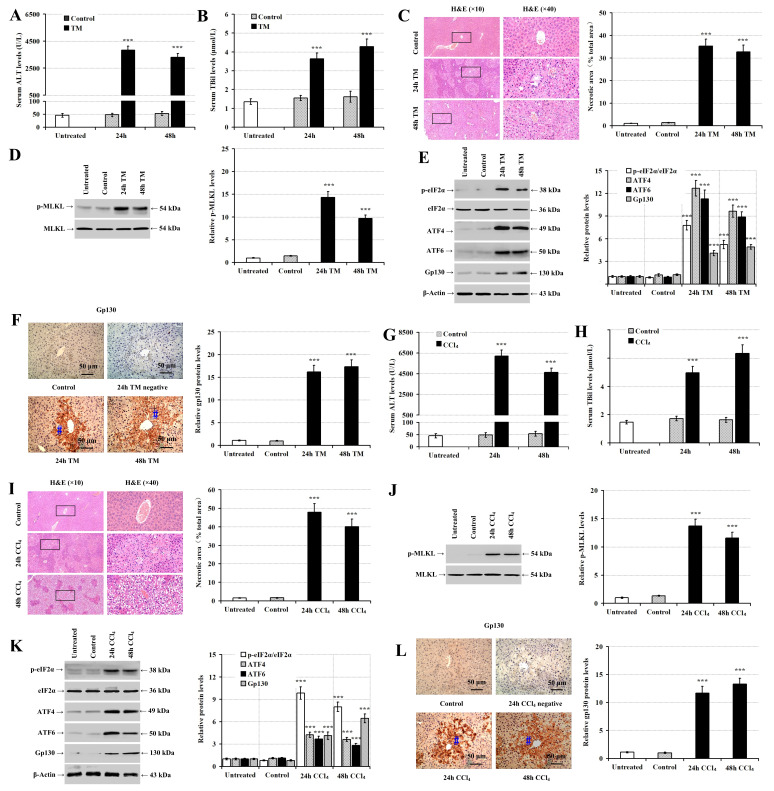
Tunicamycin (TM) and carbon tetrachloride (CCl_4_) induce endoplasmic reticulum (ER) stress and upregulate glycoprotein 130 (gp130) expression in the mouse liver. Male BALB/c mice were injected with vehicle, TM, or CCl_4_ for 24 h or 48 h (n = 12, total mice = 120). (**A**) Serum aminotransferase (ALT), and (**B**) total bilirubin (TBil) levels were determined in individual mice. (**C**) Hematoxylin and eosin (H&E) staining showed morphological changes in the mouse livers. (**D**) Western blot analysis of the relative levels of phosphorylated mixed lineage kinase domain-like pseudokinase (p-MLKL) (**E**), phosphorylated eukaryotic translation initiation factor 2α (p-eIF2α), activating transcription factor 4 (ATF4), ATF6, and gp130 protein. (**F**) Immunohistochemical staining of gp130 protein expression. The black arrows indicate positive staining. (**G**) Serum ALT and (**H**) TBil levels were determined in individual mice. (**I**) H&E staining exhibited morphological changes. (**J**) Western blot analyses of the relative levels of p-MLKL and (**K**) p-eIF2α, ATF4, ATF6, and gp130 proteins. (**L**) Immunohistochemical staining of gp130 protein expression. The black arrows indicate positive staining. Data are representative images or expressed as mean ± standard deviation (SD) of each group from four separate experiments. ***P < 0.001 vs the specified group.

Compared with the vehicle-injected and untreated controls, mice injected with TM had significantly elevated levels of serum ALT and TBil, increased necrotic areas in the liver, and enhanced MLKL phosphorylation (*P* < 0.001 for all; Figure 3A-D). This indicates that TM injection induces acute liver injury, likely resulting in hepatocyte necroptosis. Interestingly, TM injection also significantly enhanced eIF2α phosphorylation, as well as ATF4, ATF6, and gp130 protein expression (*P* < 0.001 for all; Figure 3E). Immunohistochemistry further demonstrated that TM injection significantly upregulated gp130 expression (*P* < 0.001; Figure 3F). Similarly, CCl_4_ injection induced liver injury, hepatocyte necroptosis, and upregulated p-MLKL, p-eIF2α, ATF4, ATF6, and gp130 protein expression (*P* < 0.001; Figure 3G-L). These findings demonstrate that ER stress not only induces acute liver injury but also upregulates gp130 expression in the mouse liver.

### Atf6 silencing mitigates gp130 expression in the liver tissues of CCl_4_-injected mice

**Figure 4:**
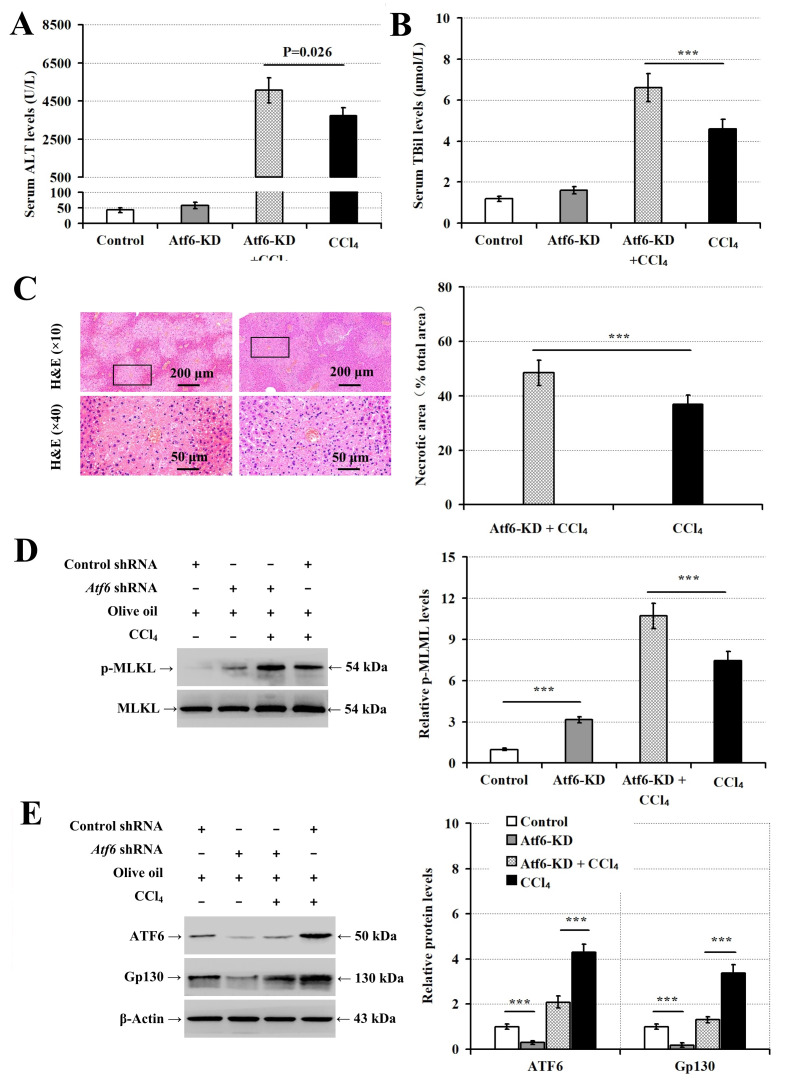
*Activating transcription factor 6* (*Atf6*) silencing reduces glycoprotein 130 (gp130) expression in the livers of carbon tetrachloride (CCl_4_)-injected mice. Male BALB/c mice were pre-treated with rAAV8 for the expression of *Atf6* shRNA or control shRNA twice, and 6 weeks later, the mice were injected with CCl_4_ or olive oil for 24 h (n = 12, total mice = 48). (**A**) Serum aminotransferase (ALT) and (**B**) total bilirubin (TBil) levels were determined in individual mice. (**C**) Hematoxylin and eosin (H&E) staining showed necrotic regions in the mouse livers. (**D**) Western blot analyses of the relative levels of phosphorylated mixed lineage kinase domain-like pseudokinase (p-MLKL), (**E**) ATF6, and gp130 proteins in the mouse livers. Data are representative images or expressed as mean ± standard deviation (SD) of each group from three separate experiments. ***P < 0.001 vs the specified group.

First, *Atf6* silencing alone did not significantly change the levels of serum ALT and TBil, but did further significantly increase the levels of serum ALT and TBil in the mice injected with CCl_4_ (*P* = 0.026 and *P* < 0.001, respectively; Figure 4A and B). This suggests that *Atf6* silencing deteriorates CCl_4_-damaged liver function. In parallel, *Atf6* silencing significantly increased the necrotic areas (*P* < 0.001; Figure 4C) and further elevated MLKL phosphorylation (*P* < 0.001 for both; Figure 4D). More importantly, *Atf6* silencing not only significantly decreased ATF6 levels and gp130 expression, but also mitigated CCl_4_-upregulated gp130 expression (*P* < 0.001; Figure 4E). In conclusion, *Atf6* silencing deteriorates acute liver injury induced by ER stress and mitigates CCl_4_-upregulated gp130 expression. The gp130 upregulated expression by CCl_4_ partially depends on ATF6 expression.

### Gp130 silencing aggravates CCl_4_-induced hepatocyte ER stress and necroptosis in mice

**Figure 5:**
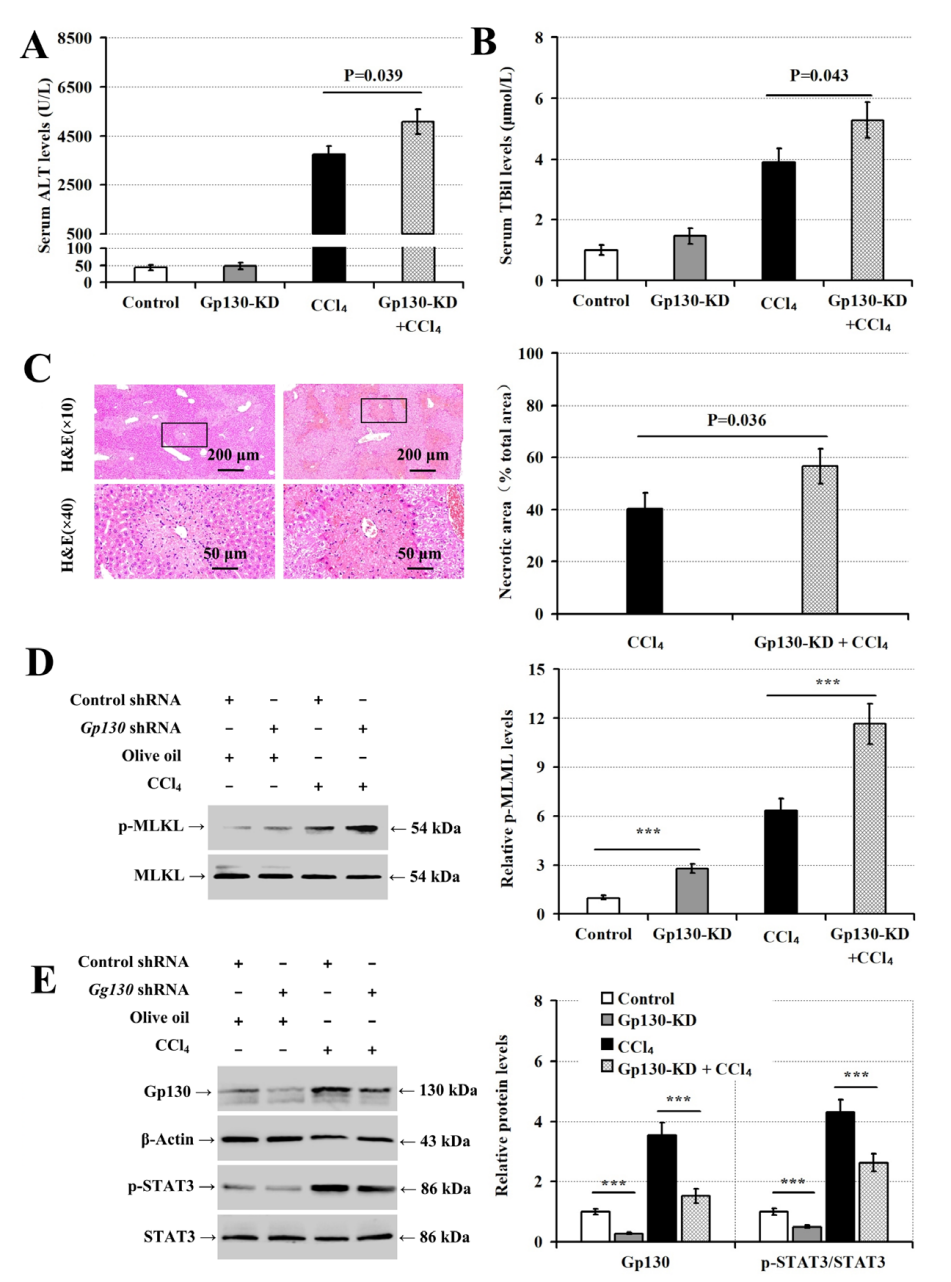
*Glycoprotein 130* (*Gp130*) silencing deteriorates carbon tetrachloride (CCl_4_)-induced hepatocyte necroptosis and endoplasmic reticulum (ER) stress in mice. Male BALB/c mice were pretreated with rAAV8 for the expression of *Gp130* shRNA or control shRNA twice, and 6 weeks later, were injected with CCl_4_ or vehicle olive oil for 36 h (n = 12, total mice = 48). (**A**) Serum aminotransferase (ALT) and (**B**) total bilirubin (TBil) levels were determined in individual mice. (**C**) Hematoxylin and eosin (H&E) staining showed necrotic areas in the mouse livers. (**D**) Western blot analyses of the relative levels of phosphorylated mixed lineage kinase domain-like pseudokinase (p-MLKL), phosphorylated signal transducer and activator of transcription 3 (p-STAT3), and (**F**) CHOP and caspase-12 proteins in the mouse livers. Data are representative images or expressed as mean ± standard deviation (SD) of each group from four separate experiments. ***P < 0.001 vs the specified group.

*Gp130* silencing did not significantly alter the levels of serum ALT and TBil in control mice, but it did significantly increase serum ALT and TBil levels in CCl_4_-injected mice (*P* = 0.039 and *P* = 0.043, respectively; Figure 5A and B). Furthermore, *Gp130* silencing significantly increased the necrotic areas in the livers of CCl_4_-injected mice (*P* = 0.036; Figure 5C) and enhanced MLKL phosphorylation in the livers of both control and CCl_4_-injected mice (*P* < 0.001 for both; Figure 5D). These findings indicate that gp130 may protect from CCl_4_-induced hepatocyte necrosis, particularly from necroptosis in mice. In addition, *Gp130* silencing significantly decreased gp130 levels and STAT3 expression, as well as STAT3 phosphorylation, in both control and CCl_4_-injected mice (*P* < 0.001 for all; Figure 5E). Finally, it dramatically increased CHOP levels and caspase-12 expression in CCl_4_-injected mice (*P* < 0.001, for both; Figure 5F). Together, these data indicate that *Gp130* silencing attenuates gp130/STAT3 signaling, and deteriorates CCl_4_-induced ER stress, hepatocyte necroptosis, and acute liver injury in mice.

## Discussion

In this study, ER stress upregulated gp130 protein expression in hepatocytes during acute liver injury. Furthermore, *ATF6* silencing aggravated ER stress-mediated hepatocyte necroptosis, and mitigated gp130 expression both *in vitro* and *in vivo*. Apparently, ER stress enhanced gp130 expression in hepatocytes, partially depending on ATF6. Interestingly, *Gp130* silencing also mitigated CCl_4_-induced STAT3 phosphorylation, as well as aggravated liver injury, hepatocyte necroptosis, and ER stress. This suggests that increased gp130 expression may protect hepatocytes from necroptosis by enhancing STAT3 activation. Therefore, hepatocyte ATF6/gp130/STAT3 signaling mitigates necroptosis during acute liver injury and may relieve ER stress.

Gp130 expression is upregulated in various types of tumors and non-neoplastic liver diseases (39,40). It can be regulated by multiple factors (41). First, gp130 binds cytokines and other molecules to transmit signals, a process leading to the endocytosis of gp130 and its ligand to avoid its sustained activation in a specific sequence-dependent manner (42,43). In addition, the stability of gp130 activity is regulated by its phosphorylation, ubiquitination, and various post-transcriptional modifications as gp130 can be degraded in the lysosomal or proteasome pathway (44,45). Moreover, ER stress can downregulate protein synthesis by enhancing PERK/eIF2α/ATF4 signaling, IRE1 endonuclease activity, and ERAD (46). Given that gp130 is a transmembrane protein, its synthesis requires the ER (47). Hence, ER stress may inhibit gp130 expression. However, our results indicated that ER stress upregulated gp130 expression in human hepatocyte cell lines and mouse livers. The differences in these findings may stem from different experimental conditions.

ER stress is mediated by PERK/eIF2α/ATF4, ATF6, and IRE1 signaling (48). PERK phosphorylates the serine Ser51 site of eIF2α, which acts as a competitive inhibitor of eIF2B and consequently inhibits the overall protein translation in the cell, thereby reducing the ER burden (49). At the same time, eIF2α can selectively initiate the expression of ATF4, which is a transcription factor, and can further induce GRP78 and CHOP expression to positively regulate ER stress (50,51). However, in our study, eIF2α phosphorylation, triggered by ER stress during acute liver injury, did not inhibit gp130 expression, and *ATF4* silencing did not significantly alter TG-upregulated gp130 expression in LO2 cells. This indicates that gp130 expression upregulated by ER stress is independent of ATF4 activity in hepatocytes.

ER stress can induce ATF6 in the Golgi apparatus to enter the nucleus, thereby promoting the expression of ER-related degradation proteins, which degrade unfolding proteins by binding to the ER stress response elements (ERSE-I, ERSE-II), UPR elements, and cAMP response elements in the promoters (52,53). ATF6 activity determines the cell fate decision between survival and death (54). In this study, *ATF6* silencing mitigated gp130 expression upregulated by ER stress in human hepatocytes and mouse livers. Furthermore, bioinformatic analysis using JASPAR found two binding sites of ATF6 with a relative score of >0.75 within the *GP130* promoter. These findings suggest that ATF6 may directly enhance *GP130* transcription during ER stress.

Gp130/STAT3 signaling is crucial for the pathogenesis of several liver diseases (55). Hepatocyte-specific gp130 knockout mice are prone to acute liver injury (56). Moreover, inhibition of IL-6/gp130 signaling can benefit patients with liver cancer (57,58). This implies that elevated gp130 expression may mitigate liver injury. In contrast, IL-6/gp130 signaling may aggravate liver damage in autoimmune hepatitis (59). The activation of gp130/JAK can activate STAT3, which modulates TNF-α, RIP1, and RIP3 expression to regulate necroptosis. We found that *gp130* silencing decreased CCl_4_-induced STAT3 phosphorylation, and aggravated liver injury, hepatocyte necroptosis, and ER stress. These findings suggest that ER stress upregulates gp130 expression in liver injury, which may lead to the induction of IL-6 and other signals in hepatocytes, and mitigate hepatocyte necroptosis. *Gp130* silencing upregulated CHOP and caspase-12 expression in the livers of CCl_4_-injected mice, a finding that suggests that the mechanisms by which gp130/STAT3 signaling alleviates necroptosis might be related to negative regulation of ER stress. Of course, previous studies have found that STAT3 signaling can reduce ER stress and necroptosis (60-62).

ER stress is hepatocytes’ defense mechanism. It can downregulate overall cellular protein synthesis but upregulate the expression of molecular chaperones by activating the ATF6, PERK, and IRE1 pathways, thus promoting hepatocyte homeostasis and cell survival. Furthermore, ER stress can modulate necroptosis by regulating the expression of RIP1, RIP3, MLKL, TNF-α, and nuclear factor kappa B (NF-κB) (63,64). It can release calcium from the ER cavity to the cytoplasm, thereby increasing the calcium concentrations in the cytoplasm and enhancing necroptosis through CaMKII/RIP1 signaling (65). ER stress can also activate NF-κB to enhance TNF-α expression and necroptosis (66-68). Our previous studies have shown that ER stress enhances hepatocyte necroptosis during acute liver injury (32) and activates ATF6 to partially alleviate hepatocyte necroptosis (69). ATF6 is an ER stress sensor and transcription factor. It regulates ER stress response by regulating the expression of different downstream target signals, promoting the homeostasis of the cell microenvironment and affecting necroptosis. In our study, ATF6 alleviated necroptosis through upregulating AFP in liver injury (70). However, it also upregulated RIP3 expression, which did not alleviate necroptosis of liver cells (69). Therefore, although ATF6 alleviates hepatocyte necroptosis during liver injury, its specific regulatory effect is extremely complex and remains to be investigated.

This study discovered that Gp130/STAT3 signaling in hepatocytes has a protective effect on the liver. However, in clinical practice, IL-6 treatment for liver damage did not achieve the expected effect. Conversely, inhibiting the activity of Gp130/STAT3 signaling has been successfully applied in the treatment of inflammatory diseases in clinical practice. This suggests that the contribution of Gp130/STAT3 signaling to the progression of liver damage in different cells may be inconsistent or even damaging. For example, the activation of Gp130/STAT3 signaling in Kupffer cells may enhance their pro-inflammatory responses and aggravate liver damage. Therefore, further research on the specific roles of the Gp130/STAT3 signaling in different cells during liver damage is needed. Also, interventions to enhance Gp130/STAT3 activity in hepatocytes while reducing it activity in pro-inflammatory cells (such as Kupffer cells) or fibrosis-promoting cells (such as hepatic stellate cells) may have more significant clinical translational implications. Additionally, the regulation of Gp130 by ATF6 should be confirmed by dual fluorescent reporter gene detection results and more *in vitro* experiments, such as primary liver cell experiments.

**Figure 6:**
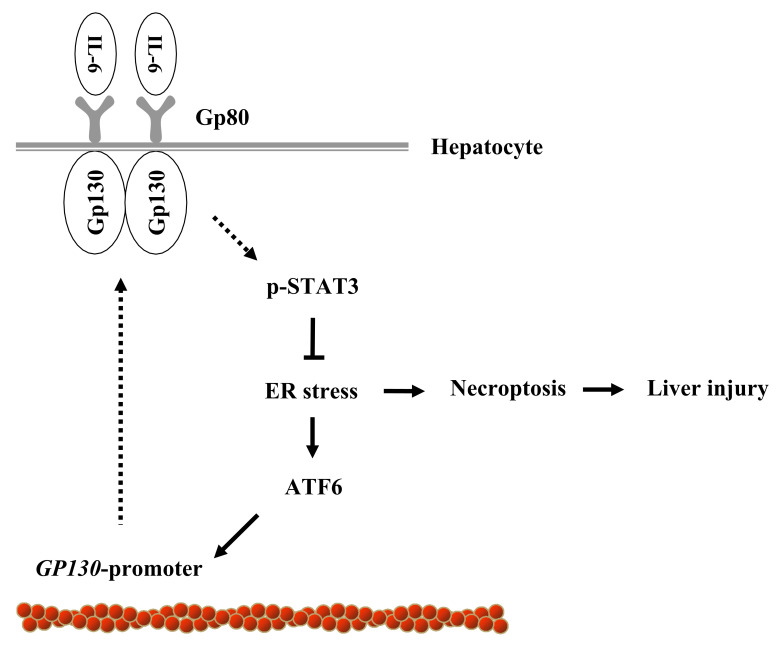
The regulation of hepatocyte necroptosis by the gp130/STAT3-ER stress axis during acute liver injury. Endoplasmic reticulum (ER) stress upregulates activating transcription factor 6 (ATF6) expression, which enhances glycoprotein 130 (gp130) expression during acute liver injury. The upregulated gp130, through activation of the signal transducer and activator of transcription 3 (STAT3) signaling, mitigates ER stress-induced hepatocyte necroptosis, thereby reducing liver injury.

In conclusion, our data indicate that gp130/STAT3 signaling modulated ER stress during liver injury, which reduced hepatocyte necroptosis. ER stress upregulated gp130 expression by activating ATF6 in hepatocytes. The upregulated gp130 expression increased STAT3 activation and mitigated hepatocyte necroptosis and liver injury, which may be related to a negative feedback alleviating ER stress (Figure 6). Therefore, our findings may provide new insights into anti-liver injury responses during ER stress-induced acute liver injury.
